# Targeting cholesterol synthesis increases chemoimmuno-sensitivity in chronic lymphocytic leukemia cells

**DOI:** 10.1186/2162-3619-3-24

**Published:** 2014-09-26

**Authors:** Indira Benakanakere, Tyler Johnson, Richard Sleightholm, Virgilio Villeda, Monika Arya, Ravi Bobba, Carl Freter, Chunfa Huang

**Affiliations:** 1 Division of Hematology/Oncology, Department of Medicine, School of Medicine, University of Missouri, Columbia, MO 65212, USA; 2 Cancer Center, Saint Louis University, Saint Louis, MO 63110, USA; 3 Division of Hematology/Oncology, Department of Internal Medicine School of Medicine, and Cancer Center, Saint Louis University, 3655 Vista Avenue, St. Louis, MO 63110-2539, USA

**Keywords:** Fludarabine, Rituximab, Cholesterol lowering agent, Chronic lymphocytic leukemia

## Abstract

**Background:**

Cholesterol plays an important role in cancer development, drug resistance and chemoimmuno-sensitivity. Statins, cholesterol lowering drugs, can induce apoptosis, but also negatively interfere with CD-20 and rituximab-mediated activity. Our goal is to identify the alternative targets that could reduce cholesterol levels but do not interfere with CD-20 in chemo immunotherapy of chronic lymphocytic leukemia (CLL).

**Methods:**

MEC-2 cells, a CLL cell line, and the peripheral blood mononuclear cells (PBMCs) from CLL patients were treated with cholesterol lowering agents, and analyzed the effect of these agents on cholesterol levels, CD-20 expression and distribution, and cell viability in the presence or absence of fludarabine, rituximab or their combinations.

**Results:**

We found that MEC-2 cells treated with cholesterol lowering agents (BIBB-515, YM-53601 or TAK-475) reduced 20% of total cellular cholesterol levels, but also significantly promoted CD-20 surface expression. Furthermore, treatment of cells with fludarabine, rituximab or their combinations in the presence of BIBB-515, YM-53601 or TAK-475 enhanced MEC-2 cell chemoimmuno-sensitivity measured by cell viability. More importantly, these cholesterol lowering agents also significantly enhanced chemoimmuno-sensitivity of the PBMCs from CLL patients.

**Conclusion:**

Our data demonstrate that BIBB-515, YM53601 and TAK-475 render chemoimmuno-therapy resistant MEC-2 cells sensitive to chemoimmuno-therapy and enhance CLL cell chemoimmuno-sensitivity without CD-20 epitope presentation or its downstream signaling. These results provide a novel strategy which could be applied to CLL treatment.

## Background

Chronic lymphocytic leukemia (CLL) is the most prevalent hematologic malignancy affecting Caucasian adults in Western countries. In the United States, about 15,000 new cases are diagnosed every year [1]. CLL is characterized by the accumulation of mature CD5, CD19, and CD23 B-lymphocytes in peripheral blood, bone marrow, lymph nodes and spleen [2]. The disease typically occurs in elderly patients (the average age at the time of diagnosis is around 72 years). Overall survival at 5 and 10 years ranges between 87% and 73% for low-risk patients and 29% and 16% for high risk patients [3]. CLL is widely heterogeneous in terms of progression, therapeutic response and outcome [4]. Approximately one-third of patients with CLL survive many years without requiring treatment, whereas others need multiple therapies early in the course of the disease [5]. Although the entire pathogenesis of CLL has not been elucidated, the widely held concept is that CLL is associated with a defective regulation of apoptosis, rather than uncontrolled cell proliferation [6].

Over the last three decades, chlorambucil has been the mainstay for the treatment of CLL; however, the complete response rate with chlorambucil is only about 10% [7-9]. Purine analogues, particularly fludarabine, are effective agents to treat CLL, resulting in a higher complete response rate than chlorambucil or alkylating-based chemotherapies (20-30% versus 10%) and a longer disease-free interval; survival, however, is not prolonged [10]. Rituximab and alemtuzumab also result in a higher complete response rate than chlorambucil in chemonaïve patients [11,12]. Recent advances have now moved away from mono-therapy to combination including the chemoimmuno-therapy [13-17]. The combination of fludarabine, cyclophosphamide and rituximab treatment achieves complete responses in only 24% to 39% of patients [15,16]. Bendamustine, an alkylating agent, plus rituximab is the other commonly used regimen for CLL, achieving a response rate of 88% and a complete response rate of 26% [7]. In spite of some progress in therapy, CLL is still considered an incurable disease because most patients generally relapse and eventually develop drug-resistance [4]. It is clear that new approaches to overcoming drug-resistance and potentiating the action of conventional therapy are urgently needed.

Cholesterol contributes to chemotherapy resistance in hepatocellular carcinoma [18], breast cancer cells [19] and prostate cancer cells [20]. Statins are inhibitors of 3-hydroxy-3-methylglutaryl-coenzyme A (HMG-CoA) reductase which catalyzes the conversion of HMG-CoA into mevalonate, a rate-limiting step in the cholesterol biosynthetic pathway [21]. Statins are commonly prescribed medications that lower serum cholesterol and prevent cardiovascular diseases [22,23]. Beyond their cholesterol-lowering properties, statins have also been shown to induce apoptosis through extrinsic and intrinsic pathways [24-26], and exhibit important antitumor activity in colorectal cancer, breast cancer, lung cancer, prostate cancer, pancreatic cancer and many other malignancies [27-29]. Although statins can induce apoptosis of acute myelogenous leukemia cell lines [30] and CLL cells [31], they are unsuitable for CLL therapy because statins interfere with the detection of CD-20 and impair rituximab-mediated complement-dependent cytotoxicity [32].

To avoid the negative interference of statins with CD-20 molecules and rituximab-mediated activity and to explore whether cholesterol levels contribute to chemoimmuno-sensitivity and drug-resistance of cancer cells, we chose several agents that inhibit squalene synthase or oxidosqualene cyclase, downstream steps in the cholesterol biosynthetic pathway, and used a CLL cell line (MEC-2) and the peripheral blood mononuclear cells (PBMCs) from CLL patients to investigate the effect of cholesterol lowering agents on the cholesterol levels, CD-20 expression and distribution, and cell viability with fludarabine, rituximab or their combinations. Our results indicate that the inhibitors of squalene synthase or oxidosqualene cyclase can significantly enhance CLL cell chemoimmuno-sensitivity.

## Results

### Effect of chemoimmuno-therapeutic drugs in MEC-2 cell viability and apoptosis

**Figure 1:**
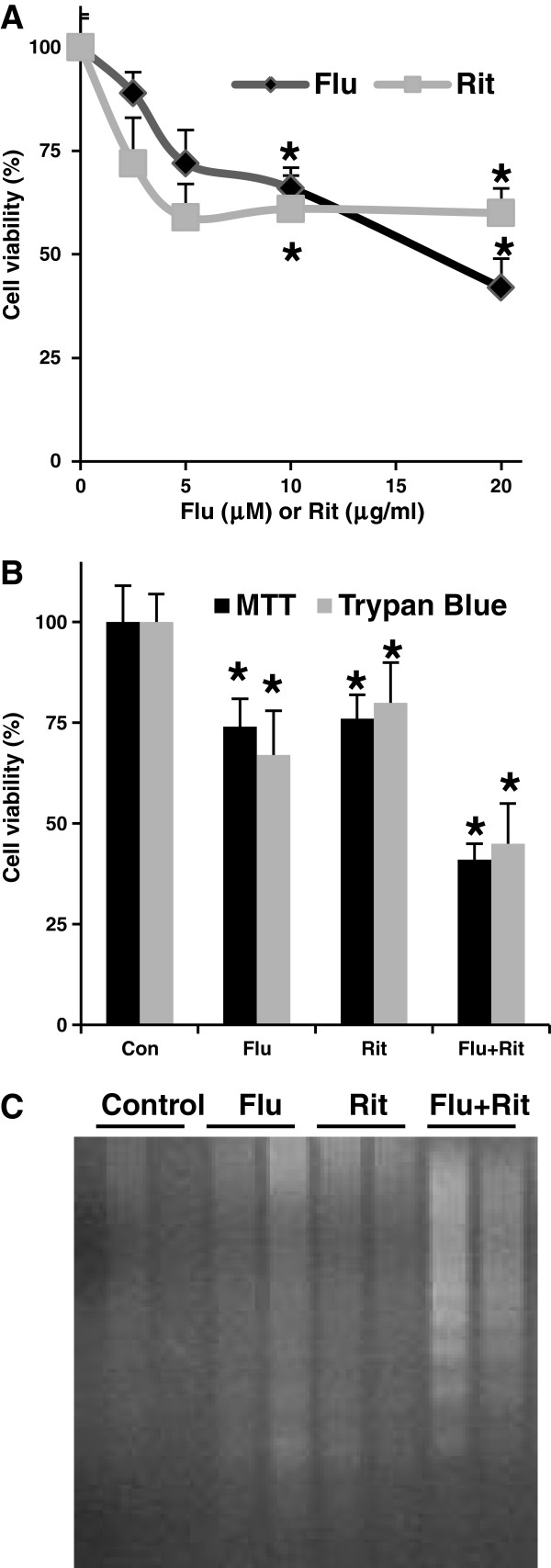
**Effect of chemoimmuno-therapeutic drugs on MEC-2 cell viability and apoptosis. A)** MEC-2 cells were treated with different concentration of fludarabine (Flu) or rituximab (Rit) (n = 16) for 3 days, and cell viability was analyzed by MTT assay. **B)** MEC-2 cells were treated with 10 μM fludarabine, 10 μg/ml rituximab or their combinations for 3 days, and analyzed cell viability by either MTT assay (n = 16) or Trypan blue staining (n = 4) and DNA fragmentation **(C)**. The DNA fragmentation represents three experiments performed with duplicate samples. The values of the treated groups were statistically different from the untreated control group. Con: control. *P < 0.05. **P < 0.01.

To investigate whether chemoimmuno-sensitivity of CLL cells could be altered, we chose MEC-2 cells, a fludarabine- and rituximab-insensitive CLL cell line. Cell viability, which indicates complement-dependent cytotoxicity that contributes significantly to clinical efficacy, was assessed by the MTT assay or Trypan blue staining after treatment. Figure 1A illustrates the dose effect of either fludarabine or rituximab on MEC-2 cell viability. At lower concentrations (<10 μM fludarabine or 10 μg/ml rituximab), fludarabine and rituximab have similar patterns in response to reduce cell viability. Higher concentrations demonstrate fludarabine cytotoxicity. Two different methods (MTT assay and Trypan blue staining) show similar results with exposure of MEC-2 cells to 10 μM fludarabine, 10 μg/ml rituximab or their combinations for 3 days. They caused about 28%, 25% or 50% reduction of cell viability, respectively (Figure 1B). To further determine whether the reduction of cell viability in drug-treated cells is due to cell growth arrest or apoptosis, we analyzed DNA fragmentation as a result of the signature events of apoptosis. Figure 1C clearly shows that treatment of MEC-2 cells with the chemoimmuno-therapeutic drugs increases DNA fragmentation. These results demonstrate that chemo- or immuno-therapeutic drugs induce MEC-2 apoptosis.

### Lovastatin enhances chemo-sensitivity in fludarabine-treated MEC-2 cells

**Figure 2:**
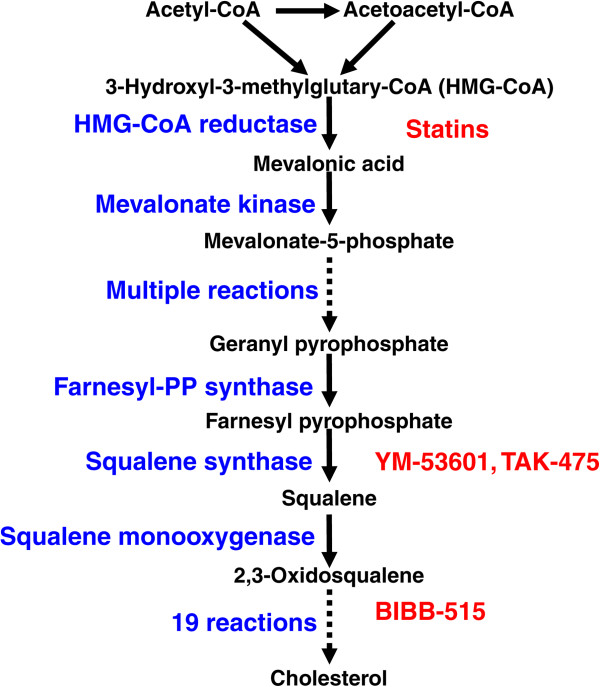
Cholesterol biosynthetic pathway and the key inhibitors.

**Figure 3:**
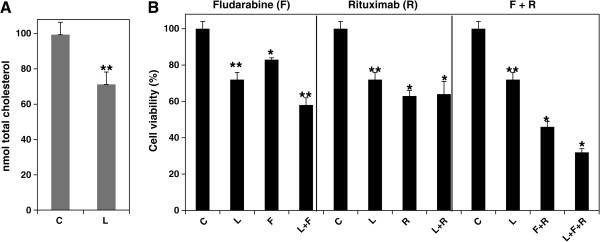
**Lovastatin lowers cellular cholesterol and enhances chemo-sensitivity. A)** MEC-2 cells were treated with 5 μM lovastatin (L) for 3 days, the samples were extracted and analyzed for total cellular cholesterol (n = 5). C, control (no lovastatin). **B)** MEC-2 cells were treated with 10 μM fludarabine (F), 10 μg/ml rituximab (R) or their combinations (F + R) in the presence or absence of 5 μM lovastatin for 3 days, and analyzed cell viability by MTT assay (n = 16). The values of lovastatin treatment were statistically different from the controls. *P < 0.05. **P < 0.01.

Earlier studies indicated that cholesterol biosynthesis (Figure 2) is mandatory for cellular growth and has been implicated in various aspects of tumor development and progression [33-35]. Certain classes of drugs, such as statins, inhibit mevalonate metabolism and exhibit growth inhibitory and pro-apoptotic properties as well as antitumor activity [27-29,36]. Next, we tested the effect of lovastatin on cholesterol metabolism and cell viability in chemoimmuno-therapeutic drug-treated MEC-2 cells. Figure 3A shows that incubation with 5 μM lovastatin for 3 days reduced cellular cholesterol levels by 30%. In cell treated with lovastatin, fludarabine and lovastatin plus fludarabine, cell viability was 75, 80, and 57% of control, respectively. However, lovastatin had little effect in the cells treated with rituximab alone (Figure 3B, middle panel). The results indicate that lovastatin may interfere with CD-20 and rituximab-mediated activity. One recent report showed that statins induce conformational changes of CD20 and impair rituximab-mediated complement-dependent cytotoxicity [32], consistent with our findings.

### Effect of BIBB-515 and YM-53601 on MEC-2 cell chemoimmuno-sensitivity

**Figure 4:**
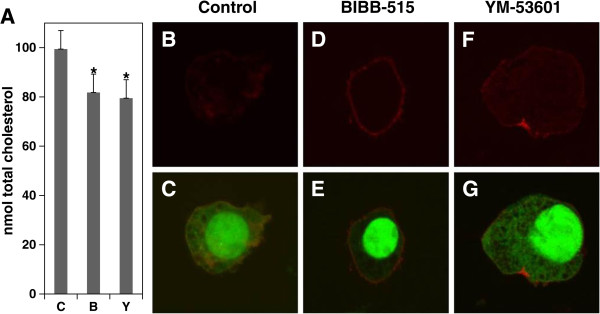
**The influence of BIBB-515 and YM-53601 on cholesterol levels and CD20 expression in MEC-2 cells.** MEC-2 cells were treated with 10 μM either BIBB-515 (B) or YM-53601 (Y) for 3 days. **A)** The cells were extracted and analyzed for total cellular cholesterol. The values of treated with BIBB-515 or YM-53601 samples were statistically different from the control (C, without BIBB-515 or YM-53601) (n = 5). *P < 0.05. **B-G)** The cells were stained for 1 hour with anti-CD-20 APC antibody and cholera toxin-B FITC, and observed and photographed by confocal microscope. The results represent three experiments performed with duplicate samples.

**Figure 5:**
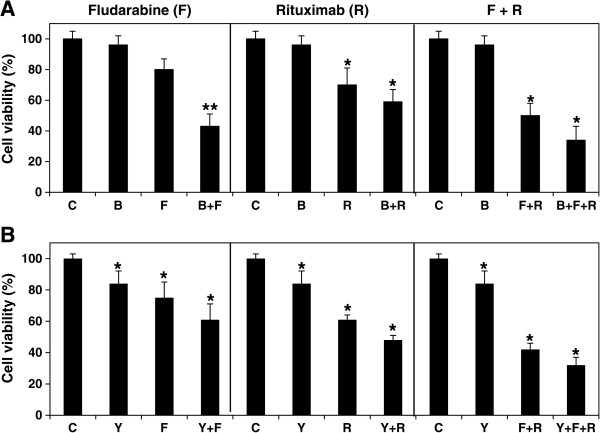
**Effect of BIBB-515 and YM-53601 on chemoimmuno-sensitivity.** MEC-2 cells were treated with 10 μM fludarabine (F), 10 μg/ml rituximab (R) or their combinations (F + R) in the presence or absence of 10 μM either BIBB-515 (B, panel **A**) or YM-53601 (Y, panel **B**) for 3 days, and analyzed cell viability by MTT assay. The values of treated with BIBB-515 or YM-53601 samples (n = 16) were statistically different from the controls (C). *P < 0.05. **P < 0.01.

To identify novel agents that not only block cholesterol biosynthesis and enhance chemoimmuno-sensitivity, but also avoid the interference with CD-20 and rituximab-mediated activity, we targeted squalene synthase and oxidosqualene cyclase which are further downstream steps in cholesterol biosynthetic pathway (Figure 2). YM-53601 is a novel squalene synthase inhibitor that reduces plasma cholesterol and triglyceride levels in several animal species [37]. BIBB-515 is a selective inhibitor of 2,3-oxidosqualene cyclase and inhibits LDL production in both normolipemic and hyperlipemic hamsters [38]. Figure 4A shows that treatment of MEC-2 cells with either BIBB-515 or YM-53601 for 3 days resulted in about 20% decrease in total cellular cholesterol. By confocal microscopy, we also analyzed the expression and distribution of CD-20 in the cells. As shown in Figure 4D, E, F and G, both BIBB-515 and YM-53601 slightly intensified CD-20 immunostaining, and dramatically increased CD-20 membrane association which would, in turn, lead to increased signaling through CD-20 to downstream pathways. Further studies indicated that either BIBB-515 or YM-53601 enhanced chemoimmuno-sensitivity in the cells treated with fludarabine, rituximab or their combinations. Cell viability was reduced from 80% to 43% in BIBB-515 (Figure 5A left) and from 75% to 61% in YM-53601 (Figure 5B left) with fludarabine treatment alone; from 70% to 59% in BIBB-515 (Figure 5A middle) and from 61% to 48% in YM53601 (Figure 5B middle) with rituximab treatment alone; and from 50% to 34% in BIBB-515 (Figure 5A right) and from 42% to 32% in YM-53601 (Figure 5B right) with their combination treatment. The data demonstrates that either BIBB-515 or YM-53601 can lower cellular cholesterol levels, up-regulate CD-20 membrane expression and enhance cell chemoimmuno-sensitivity.

### TAK-475 enhances chemoimmuno-sensitivity in MEC-2 cells

**Figure 6:**
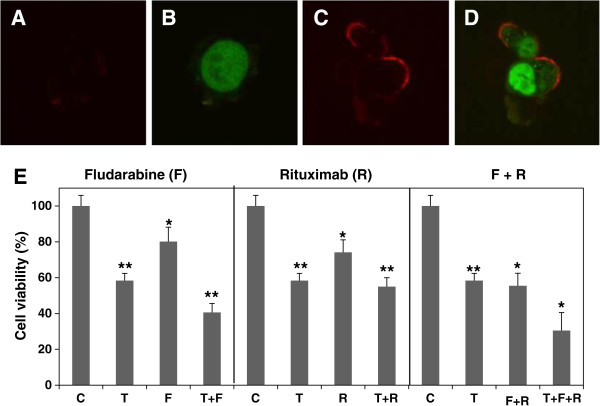
**Effect of TAK-475 on CD-20 expression and chemoimmuno-sensitivity.** MEC-2 cells were treated with 10 μM TAK-475 (T) for 3 days, processed for double immunostaining, and photographed by confocal microscope **(A-D)**. The results represent three experiments performed with duplicate samples. The cells were also treated with 10 μM fludarabine (F), 10 μg/ml rituximab (R) or their combinations (F + R) in the presence or absence of 10 μM TAK-475 for 3 days, and then analyzed cell viability by MTT assay **(E)**. The values of treated with TAK-475 samples (n = 16) were statistically different from the controls (C). *P < 0.05. **P < 0.01.

TAK-475 (Lapaquistat) is another squalene synthase inhibitor that has been used as a cholesterol lowering drug in Phase III clinical trials in Europe and the United States [39]. To investigate the effect of TAK-475 on chemoimmuno-sensitivity, we firstly treated MEC-2 cells with 10 μM TAK-475 for 3 days, and explored its effect on CD-20 expression and distribution. As shown in Figure 6, both CD-20 staining and membrane association are significantly increase in TAK-475 treated (Figure 6C and D) compared with those controls (Figure 6A and B). Next, we analyze the effect of TAK-475 on MEC-2 cell chemoimmuno-sensitivity. As shown in Figure 6E, TAK-475 enhances chemoimmuno-sensitivity of fludarabine, rituximab and their combinations. With TAK-475, cell viability was decreased from 72% to 40% with fludarabine treatment alone, from 80% to 59% with rituximab treatment alone, and from 41% to 26% with their combination treatment.

### BIBB-515 and TAK-475 enhance chemoimmuno-sensitivity in the PBMCs

**Figure 7:**
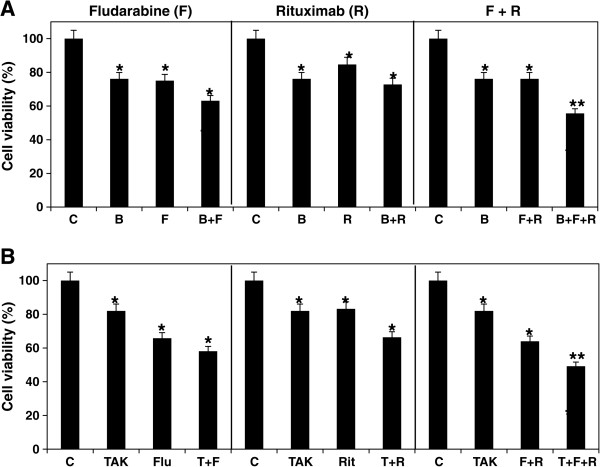
**Effect of BIBB-515 and TAK-475 on chemoimmuno-sensitivity in the PBMCs from CLL patients.** The PBMCs were treated with or without 10 μM fludarabine (F), 10 μg/ml rituximab (R) or their combinations (F + R) in the presence or absence of 10 μM either BIBB-515 (B, panel **A)** or TAK-475 (T, panel **B)** for 3 days, and cell viability was determined by Trypan blue live-dead cell staining. The values of treated with BIBB-515 (n = 10) or TAK-475 (n = 6) samples were statistically different from the controls (C). *P < 0.05. **P < 0.01.

Our results show that cholesterol lowering agents enhance chemoimmuno-sensitivity in MEC-2 cells. To investigate the potential cholesterol lowering agents for CLL chemoimmuno-therapy, we isolated the PBMCs from CLL patients, treated the cells with 10 μM fludarabine, 10 μg/ml rituximab, or their combinations in the presence or absence of either 10 μM BIBB-515 or TAK-475 for 3 days, and determined cell viability by Trypan blue staining. With BIBB-515, cell viability was reduced from 75% to 63% with fludarabine alone, from 85% to 73% with rituximab alone, and from 76% to 57% with their combinations (Figure 7A). With TAK-475, cell viability was reduced from 66% to 58% with fludarabine alone, from 82% to 66% with rituximab alone, and from 64% to 49% with their combinations (Figure 7B). These results demonstrate that either BIBB-515 or TAK-475 enhances chemoimmuno-sensitivity in the PBMCs from CLL patients.

## Discussion

Cholesterol plays an essential role in the stability and architecture of the plasma membrane and in the involvement of vesicle traffic and receptor signaling [40,41]. Emerging data from profiling of cancer tissues and animal models [42-44] as well as *in vitro* cancer cell lines [45] demonstrate that cholesterol is capable of regulating cell proliferation, migration, and signaling pathways in carcinogenesis, tumor development and chemotherapy resistance. Recognizing cholesterol as an important factor contributing to cancer development, many researchers focus on manipulating cholesterol metabolism as novel targets for cancer therapy [18-25]. Statins, cholesterol lowering agents, inhibit mevalonate metabolism and exhibit antitumor effects against various cancer cell lines [27-29]. Using lovastatin, we reported here that lowering cholesterol exhibited increased chemosensitivity in fludarabine-treated MEC-2 cells, but had no effect on immunotherapy in rituximab-treated cells (Figure 3). Earlier data from *in vitro*[32] and *in vivo*[46] also showed that statins do not influence clinical response and B cell depletion after rituximab treatment because statins induce conformational changes in CD-20 molecules and impair rituximab-mediated complement-dependent cytotoxicity.

CLL cell killing by rituximab requires cross linking of CD-20, which induces redistribution of CD-20 to lipid rafts. Lipid rafts are specialized microdomains of the plasma membrane that are enriched in sphingolipid and cholesterol, and play an important role in the initiation of many anticancer drug-induced signaling pathways and toxicological effects. Anticancer drugs are able to suppress cell growth and induce apoptosis of tumor cells through disrupting lipid raft integrity [32,47]. The redistribution of CD-20 may result in lipid raft disruption, activate or deactivate raft-associated proteins, such as death receptors and protein kinases in apoptotic pathway which are correlated with efficiency of complement-dependent cytotoxicity and antibody-dependent cell-mediated cytotoxicity. Rituximab and other anti-CD-20 antibodies are currently key drugs in CLL chemoimmuno-therapy. Although statins have antitumor activity, they are clearly unsuitable for enhancement of chemoimmuno-sensitivity of lymphomas and leukemias.

To search for new agents that can lower cellular cholesterol, and do not negatively interfere with CD-20 and rituximab-mediated activity, we chose three compounds that can inhibit squalene synthase (YM-53601 and TAK-475) or oxidosqualene cyclase (BIBB-515). These enzymes are further downstream steps in cholesterol biosynthetic pathway (Figure 2). By confocal microscopy, our results indicate that treatment of MEC-2 cells with YM-53601, BIBB-515 or TAK-475 did significantly up-regulate CD-20 surface expression and membrane-association (Figures 4 and 6). One recent study has shown that farnesyltransferase inhibitors may up-regulate CD-20 at mRNA and protein levels and improve anti-CD-20 monoclonal antibody-mediated activation of complement-dependent cytotoxicity [48]. Earlier reports demonstrated that cytokines (interleukin-4, granulocyte-macrophage colony-stimulating factor, or interferon-α) also increase CD-20 surface expression [46,49]. Taken together, our data and those results indicate that the agents inhibiting downstream steps of cholesterol biosynthesis and other cholesterol-associated pathways could positively regulate CD-20 expression and its antibody-mediated signaling.

Our observations indicate for the first time that BIBB-515, YM-53601 and TAK-475 significantly inhibit their respective enzymes in the pathway of cholesterol biosynthesis to lower cellular cholesterol levels, and that targeting these enzymes could positively affect CD-20 distribution and its antibody-mediated activation in contrast to statins. Lowering cellular cholesterol levels with statins has been shown to induce apoptosis, [24-26] and have important antitumor activity in some malignancies [27-29]. Our experiments demonstrate that the cholesterol lowering agents BIBB-515, YM-53601 and TAK-475 render chemoimmuno-therapy resistant cells sensitive to chemoimmuno-therapy, and enhance cell chemoimmuno-sensitivity and apoptosis (Figures 5 and 6). These results validate the concept that cholesterol reduction using enzymatic inhibitors is different to the action of statins, probably due to different mechanism of activation of CD-20 dependent pathways.

We have also observed the effect of BIBB-515 and TAK-475 on chemoimmuno-sensitivity in the PBMCs from CLL patients. These cells from different patients show differences in response to three-day chemoimmuno-therapeutic drug treatment. The PBMCs from two patients (total 14 patients) weakly responded to fludarabine, and three patients are very weak in response to rituximab; however, all of these patient cells showed a 5-30% increase of chemoimmuno-sensitivity with cholesterol lowering agents. In these patients, two patients had undergone fludarabine treatment and one had undergone a round of chlorambucil and rituximab treatment. These PBMCs also show increased chemoimmuno-sensitivity with fludarabine, rituximab or their combinations in the presence of cholesterol lowering agents. Although cells treated with multiple drugs could cause pleiotropic effects, it is important that the combinations of BIBB-515, YM-53601 or TAK-475 and chemoimmuno-therapeutic drugs are synergistic effects. Our findings regarding the role of cholesterol on chemoimmuno-therapy along with recent reports in different types of cancer cells [18-29] highlight the relevance of cholesterol modulation in cancer therapy.

Decreased cell growth by lowering cellular cholesterol levels with statins in different cancer cells have been previously shown, we demonstrate that the mechanism of cholesterol modulation by other inhibitors is important because statins negatively affect CD-20 expression and antibody-mediated cell killing through CD-20. Our data demonstrate that BIBB-515, YM-52601 and TAK-475 acting at squalene synthase or oxidosqualene cyclase reduce cellular cholesterol levels, and inhibit cell growth leaving a CD-20 mediated pathway intact. Compensatory changes in membrane lipid composition as a result of treatment with these cholesterol synthesis inhibitors are an area we are investigating. Taken together, our results demonstrate that membrane lipid alteration in cancer cells is a novel and viable approach to both cancer therapeutics and reversal of chemoimmuno-therapy resistance.

## Material and methods

### Materials

All chemicals were purchased from Sigma-Aldrich (St Louis, MO, USA) or Fisher Scientific (Pittsburgh, PA, USA) unless specified otherwise. Ficoll-Paque Plus was obtained from Amersham Biosciences (Piscataway, NJ, USA). The media for cell culture were purchased from GIBCO (Grand Island, NY, USA). The anti-CD-20 APC antibody was obtained from eBiosciences (San Diego, CA, USA). CellTiter 96® Non-Radioactive Cell Proliferation Assay kit (MTT) was purchased from Promega (Madison, WI, USA). Fludarabine was obtained from Teva Parenteral Medicines, Inc (Irvine, CA, USA). Rituximab was obtained from Genentech (South San Francisco, CA, USA). BIBB-515 and YM-53601 were purchased from Cayman Chemical Company (Ann Arbor, MI, USA). TAK-475 was obtained from Chemzon Scientific (Montreal, Quebec, Canada).

### Cell culture, PBMC isolation and cell treatment

MEC-2 cells were cultured in DMEM/F-12 media with 10% FBS. Cells were grown at 37°C in 5% CO_2_. Cholesterol lowering agents (BIBB-515, YM-53601 or TAK-475) were added to the cultures at 10 μM final concentration in the presence or absence of fludarabine (10 μM), rituximab (10 μg/ml) or their combinations for 3 days. Treated cells were used for analysis according to experimental design.

Blood was obtained from CLL patients as defined by NCI96 criteria 28 [50] following receipt of written informed consent under an IRB protocol approved by the University of Missouri-Columbia. PBMCs were isolated from whole blood immediately following donation using Ficoll density gradient centrifugation. Isolated cells were treated with test compounds as experimental designed and incubated at 37°C and 5% CO_2_ in RPMI-1640 media for 3 days. Cells were counted manually on a hemocytometer with Trypan blue solution before treatment, and at 1, 2, and 3 days after treatment to determine cell viability.

### Total cholesterol analysis

Three million MEC-2 cells in the experiments were harvested, and 6 μl of standard cholesterol (1 mg/ml of chloroform) was added to the samples. Total cholesterol was extracted by the method of Bligh and Dyer [51]. The resulting organic lower phase was withdrawn and evaporated under a stream of N_2_. One milliliter of methanolic potassium hydroxide was added to the tubes, and the samples were heated at 80°C for 1 hour. The tubes were sonicated several times during saponification. After cooling down, 2 ml of HPLC-grade water was added and the sterols were recovered by extracting the mixture 3 times with 3 ml hexane. The hexane extracts were pooled and dried down under N_2_. After complete dry, the samples were re-dissolved in 70 μl pyridine. The samples were derivatized by adding 30 μl N-trimethylsilyl-N-methyltrifluoroacetamide with trimethylchlorosilane and incubating at 50°C for 1 hour. The solvents were completely dried and re-dissolved in 75 μl of chloroform. Gas chromatography mass spectrometry (GC/MS) analysis was performed at the Kansas Lipidomics Research Center on an Agilent 6890 N GC coupled to an Agilent 5975 N quadrupole mass selective detector. The data were collected and processed with Agilent Chemstation.

### Confocal microscopy

MEC-2 cells were treated with various compounds for 3 days, washed in cold medium, and fixed with 4% paraformaldehyde in 100 mM phosphate buffer on ice. The fixed cells were washed twice with 1% BSA in PBS (BSA buffer). The cells were blocked for 15 minutes in 5% mouse serum in PBS and washed with BSA buffer. The cells were stained for 1 hour with anti-CD-20 APC antibody and cholera toxin-B FITC. Wet-mount slides were prepared, and the samples were viewed by a Zeiss 510 META laser scanning confocal microscope and photographed by LSM 4.2 software.

### Cell viability assays

MEC-2 cells were plated in 96-well assay plates at a concentration of 10,000 cells per well in 100 μl of culture medium with test compounds according to experimental designed. After adding the agents, the cells were cultured at 37°C for 3 days, and cell viability was determined using Promega’s CellTiter 96® Non-Radioactive Cell Proliferation Assay kit (MTT) according to the manufacturer’s instructions. Absorbance at 570 nm was recorded using a Biotek Synergy H4 Hybrid Reader (Winooski, VT). MEC-2 cells and PBMCs were stained with Trypan blue solution, and dead and alive cells were counted after treatment to determine cell viability.

### Measurement of DNA fragmentation

According to experimental designed, treatment of MEC-2 cells were harvested and centrifuged at 1,500 *g* for 2 min, and the pellets were resuspended in 0.5 ml of lysis buffer containing 5 mM Tris-HCl, pH 8.0, 20 mM EDTA, and 0.5% Triton X-100 and placed on ice for 15 min. The samples were then centrifuged at 12,000 *g* for 20 min, and the supernatant containing DNA cleavage products in the same amount of cellular proteins was precipitated overnight using isopropyl alcohol. The samples were centrifuged at 24,446 g for 20 min. Pellets were resuspended in Tris-EDTA buffer and digested with 0.2 mg/ml proteinase K and 1 mg/ml RNase A for 60 min at 48°C. DNA fragments were separated on a 1.5% agarose gel, visualized with ethidium bromide, and photographed using the Bio-Rad image system.

### Data analysis

Statistical analysis was done using Sigma plot 12. The difference in the mean values among treatment groups to the controls were analyzed by one way analysis of variance.

## Abbreviations

CLL: Chronic lymphocytic leukemia; PBMC: Peripheral blood mononuclear cell; HMG-CoA: Hydroxy-3-methylglutaryl-coenzyme A.

## Competing interests

The authors declare that they have no competing interests.

## Authors' contributions

IB, CF, and CH conceived the experimental design. IB, TJ, and CH performed the experiments and analyzed the data. VV, RB, MA, and RS collected the patient samples and analysis. IB, CF and CH interpreted the data. CH and CF wrote the paper. All authors read and approved the final manuscript.
